# Formation of Peptide Bound Pyrraline in the Maillard Model Systems with Different Lys-Containing Dipeptides and Tripeptides

**DOI:** 10.3390/molecules21040463

**Published:** 2016-04-07

**Authors:** Zhili Liang, Lin Li, Haiping Qi, Liting Wan, Panfu Cai, Zhenbo Xu, Bing Li

**Affiliations:** 1 School of Food Science and Engineering, South China University of Technology, Guangzhou 510640, China; zhililiang1988@gmail.com (Z.L.); oliveauspicious@sina.com (H.Q.); nbwlting@163.com (L.W.); painful_tsai@126.com (P.C.); zhenbo.xu@hotmail.com (Z.X.); 2 Guangdong Province Key Laboratory for Green Processing of Natural Products and Product Safety, South China University of Technology, Guangzhou 510640, China; 3 University Affairs Committee, Dongguan University of Technology, Dongguan 523808, China; 4 Department of Microbial Pathogenesis, Dental School, University of Maryland, Baltimore, MD 21201, USA

**Keywords:** peptide bound AGEs, pyrraline, peptide, the Maillard reaction, physicochemical properties

## Abstract

Peptide-bound advanced glycation end-products (peptide-bound AGEs) can be formed when peptides are heated with reducing saccharides. Pyrraline is the one of most commonly studied AGEs in foods, but the relative importance of the precursor peptide structure is uncertain. In the present study, model systems were prepared by heating peptides with glucose from 60 °C to 220 °C for up to 65 min, and the amounts of peptide-bound pyrraline formed were monitored to evaluate the effect of the neighboring amino acids on the peptide-bound pyrraline formation. The physico-chemical properties were introduced to explore the quantitative structure-reactivity relationships between physicochemical properties and peptide bound formation. 3-DG content in dipeptide-glucose model system was higher than that in the corresponding tripeptide-glucose model systems. Dipeptides produced higher amounts of peptide-bound pyrraline than the corresponding tripeptides. The peptide-bound pyrraline and 3-DG production were influenced by the physico-chemical properties of the side chain of amino acids adjacent to Lys in the following order: Lys-Leu/glucose > Lys-Ile/glucose > Lys-Val/ glucose > Lys-Thr/glucose > Lys-Ser/glucose > Lys-Ala/ glucose > Lys-Gly/glucose; Lys-Leu-Gly/glucose > Lys-Ile-Gly/glucose > Lys-Val-Gly/glucose > Lys-Thr-Gly/glucose > Lys-Ser-Gly/glucose > Lys-Ala-Gly/glucose > Lys-Gly-Gly/glucose. For the side chain of amino acids adjacent to Lys in dipeptides, residue volume, polarizability, molecular volume and localized electrical effect were positively related to the yield of peptide bound pyrraline, while hydrophobicity and pK_b_ were negatively related to the yield of peptide bound pyrraline. In terms of side chain of amino acid adjacent to Lys in tripeptides, a similar result was observed, except hydrophobicity was positively related to the yield of peptide bound pyrraline.

## 1. Introduction

Since 1980, advanced glycation end products (AGEs), stable amino acid derivatives of the lysine and arginine side chains of proteins and peptides, have attracted increasing attention due to their pathophysiological role in diabetes [1,2,3,4]. Modern diets are largely heat-processed and as a result contain high levels of advanced glycation end products (AGEs). Even though Ames has advocated that dietary AGEs are not harmful to human health based on the review of information obtained from animal and human studies concerning the bioavailability and metabolic fate of dietary AGEs [5], dietary advanced glycation end products (dAGEs) are still known to contribute to increasing oxidant stress and inflammation, which are linked to the recent epidemics of diabetes and cardiovascular disease [6,7]. However, no proof of the impact of individual compounds has been reported until the present time. Some kinetic studies have estimated that 10%–30% of dAGEs consumed can be absorbed intestinally and enter the circulation in the human body [8,9]. Pyrraline is one of the common AGEs, which has been detected in various food products. Up to 12.2 mg/kg pyrraline has been detected in milk products, however, in caramel candy, the pyrraline content is between 56.2 and 134.9 mg/kg protein. In the bakery products, the amounts of pyrraline have reached up to 242.6 mg/kg [10]. Pyrraline is often formed in the final stage of the Maillard reaction (“glycation”), which is characterized by covalent modification and cross-linking of amino acids, predominantly caused by reactions with 1,2-dicarbonyl compounds which originate from saccharide degradation. More specifically, it is formed by the addition of 3-deoxyglucosone (3-DG) to the amino group of the lysine side chain [11,12,13]. According to the lysine residue, pyrraline mainly exists in two forms: free-form and bound to lysine residues within peptides or proteins. In fact, the amount of free amino acids in food is always very low as compared to the amounts of peptides and proteins [14]. Therefore, it can be assumed that in foods, peptide- and protein-bound pyrraline formation derived from peptides and proteins by the Maillard reaction are much more important than the free-form pyrraline derived from free amino acids. However, due to the complexity of protein structure, it is not easy to uncover the effects of protein structure on the formation of protein-bound pyrraline. In order to gain a deeper insight into the protein-bound pyrraline formation, firstly, dipeptide- and tripeptide-bound pyrraline formation should be clearly explored.

A limited number of studies have been conducted on the reactivity of peptides in the Maillard reaction. The majority of these studies have focused on the free amino acid, glycine homopolymer in model systems representing either physiological [15,16,17] or food-related conditions [17]. Although their results can represent the reactivity of free amino acids, di-, tri- and tetrapeptides in the Maillard reaction [18], the reactivity in their case has been defined as the rate in which glucose has been converted in the early-phase of the Maillard reaction, only the Schiff base formation and Amadori rearrangement reaction have been involved, and the products at the final stage of the Maillard reaction have not been mentioned.

Consequently, the effect of structure and length of peptide on peptide-bound pyrraline formation should be taken into consideration. In addition, for the further study of the peptide-bound pyrraline formation, it is very important to profoundly understand the relationship between peptide-bound pyrraline formation and the well-characterized chemical structures of peptides. In the present study, seven different dipeptides and their corresponding tripeptides with lysine at the N-terminus (Lys-X and Lys-X-Gly, X = Ala, Gly, Ser, Ile, Leu, Thr, Val) were reacted with glucose to determine the factors that impact the peptide-bound pyrraline formation (Pyr-X and Pyr-X-Gly, X = Ala, Gly, Ser, Ile, Leu, Thr, Val). In fact, different side chains structures of these peptides were selected in order to reduce some uncertainty factors. The amino acids adjacent to Lys were varied to study the influence of the neighboring amino acid on the pyrraline production, since, theoretically, the Lys residue properties can be influenced by the neighboring amino acid. Several physico-chemical properties (*i.e.*, hydrophobicity, polarizability, residue volume, pK_b_, localized electrical effect, molecular volume) were employed to explore the mechanism(s) that lead to differences in peptide- bound pyrraline formation among different peptide-glucose model systems.

## 2. Results

### 2.1. 3-DG in the Mixtures

**Scheme 1:**
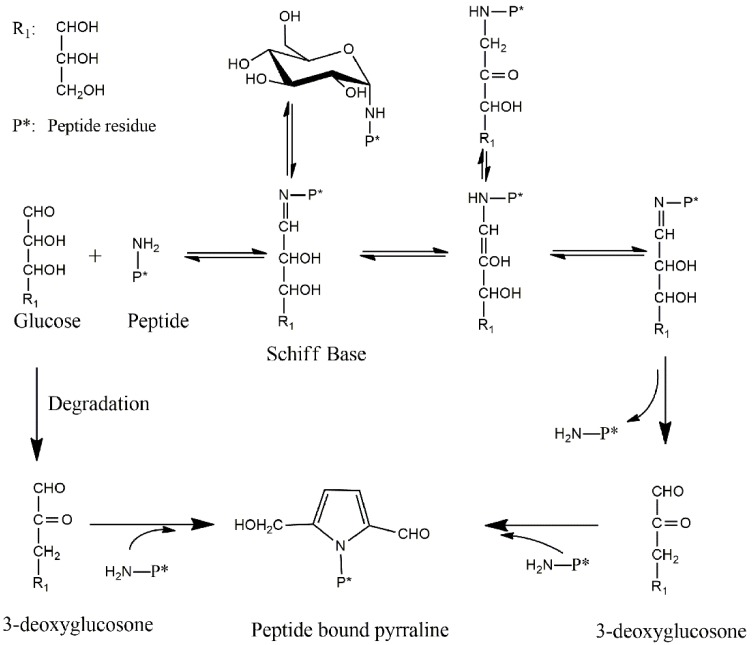
Schematic representation of the potential pathways leading to pyrraline formation during the Maillard reaction.

Two main pathways from glucose to 3-DG are shown in Scheme 1. One is the degradation from glucose without -NH_2_ residue involvement [19,20], another is the glucose fragmentation at the intermediate stage of the Maillard reaction with -NH_2_ residue involvement [21]. Therefore, it is very necessary to monitor the 3-DG concentration during pyrraline formation.

**Figure 1:**
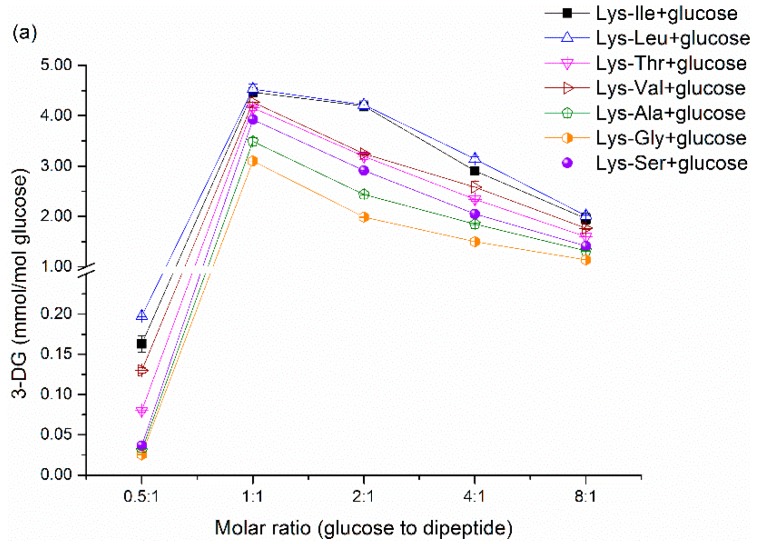

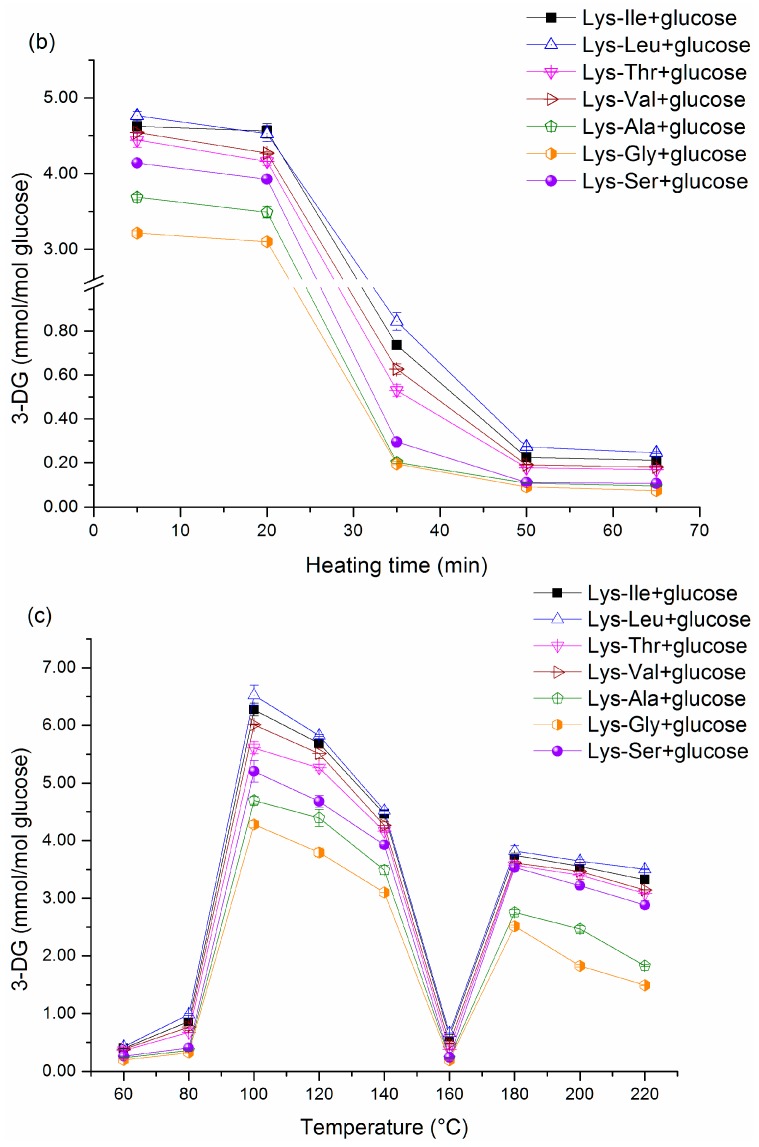
3-DG content in the heat-treated dipeptide-glucose mixtures, (**a**) dipeptide-glucose mixtures at different initial molar ratios, time 20 min, temperature 140 °C; (**b**) dipeptide-glucose mixtures at various heat-treated times, initial molar ratio 1:1, temperature 140 °C; (**c**) dipeptide-glucose mixtures at different heating temperatures, initial molar ratio 1:1, time 20 min.

**Figure 2:**
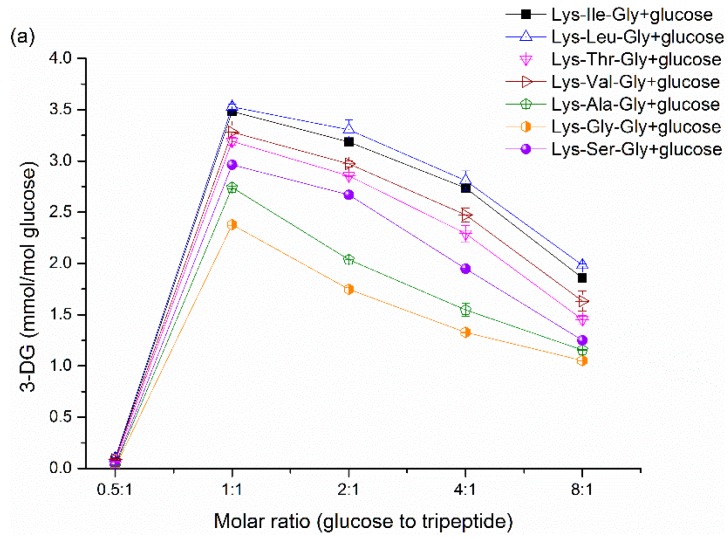

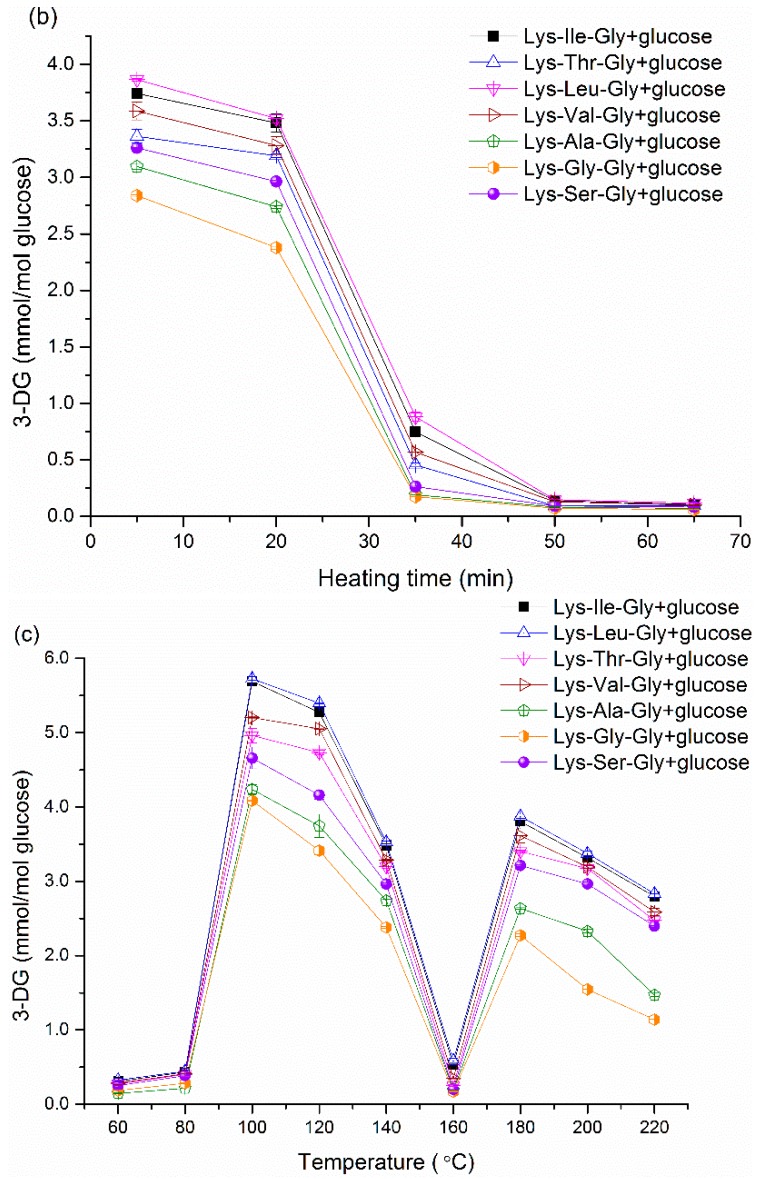
3-DG content in the heat-treated tripeptide-glucose mixtures, (**a**) tripeptide-glucose mixtures at different initial molar ratios, time 20 min, temperature 140 °C; (**b**) tripeptide-glucose mixtures at various heat-treated times, initial molar ratio 1:1, temperature 140 °C; (**c**) tripeptide-glucose mixtures at different heating temperatures, initial molar ratio 1:1, time 20 min.

There was a significant difference in 3-DG content among different dipeptide-glucose mixtures (*p* < 0.05) at the same molar ratio (except at 0.5:1, Figure 1a). 3-DG content generally decreased in the following order: Lys-Leu/glucose > Lys-Ile/glucose > Lys-Val/glucose > Lys-Thr/glucose > Lys-Ser/glucose > Lys-Ala/glucose > Lys-Gly/glucose. Interestingly, a similar result was obtained in the tripeptide-glucose model systems (Figure 2a). Moreover, as shown in Figure 1a and Figure 2a, the 3-DG content was higher in the dipeptide-glucose mixture than that in the corresponding tripeptide-glucose mixture at the same molar ratio (except at 0.5:1).

In addition, a gradual decline in 3-DG content was observed in Figure 1b and Figure 2b with prolonged heating time. There was a sharp decrease in 3-DG concentration from 20 min to 35 min, after that, no significant difference in 3-DG content was observed among the seven dipeptide-glucose mixtures, as well as tripeptide-glucose mixtures. However, at the same heating time within 30 min, 3-DG content generally decreased in the following order: Lys-Leu/glucose > Lys-Ile/glucose > Lys-Val/glucose > Lys-Thr/glucose > Lys-Ser/glucose > Lys-Ala/glucose > Lys-Gly/glucose. A similar result was also observed in the tripeptide-glucose model systems.

In terms of the thermal treatment at different temperatures, a very complicated trend in 3-DG content was observed in all peptide-glucose mixtures with rising temperatures (Figure 1c and Figure 2c). At the same heating temperature, 3-DG content in dipeptide-glucose mixtures declined in the following order: Lys-Leu/glucose > Lys-Ile/glucose > Lys-Val/glucose > Lys-Thr/glucose > Lys-Ser/glucose > Lys-Ala/glucose > Lys-Gly/glucose (significant differences except at 60, 80 and 160 °C, *p* < 0.05). Likewise, an analogous trend in 3-DG concentration was shown in tripeptide-glucose mixtures as compared to that in dipeptide-glucose mixtures (Figure 2c). Comparing Figure 1c and Figure 2c, 3-DG content in each dipeptide-glucose mixture was higher than that in the corresponding tripeptide-glucose mixture at the same heating temperature (except at 60 °C, 80 °C and 160 °C).

### 2.2. Peptide Loss in the Mixtures

The Maillard reaction can lead to the loss of amino acids, especially lysine. Since lysine is an essential amino acid, a damaging effect on the overall nutritional value of food can be attributed to the irreversible loss of lysine during the Maillard chemistry reactions [22]. In addition, because the -NH_2_ residue of lysine is a necessary factor for AGE formation in the Maillard reaction, in the present study, the peptide loss was employed as one of important indicators to monitor the extent of the Maillard reaction.

**Figure 3:**
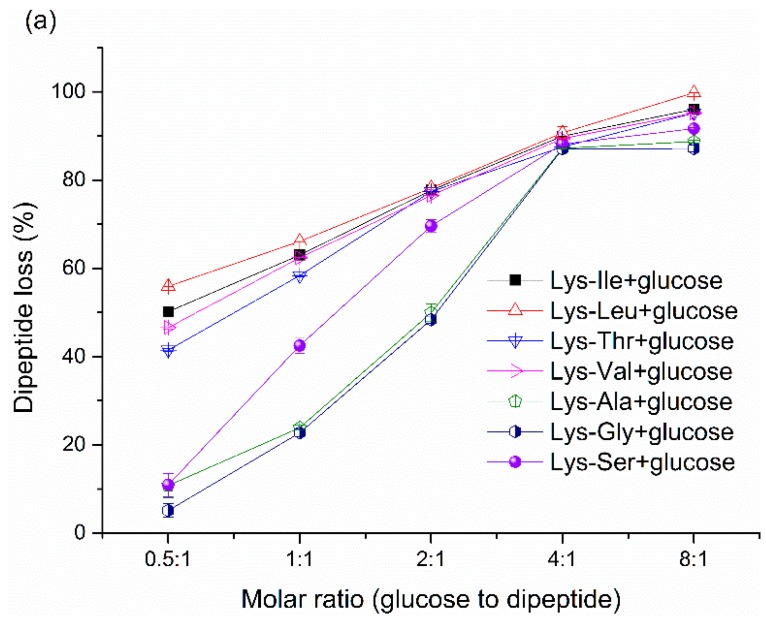

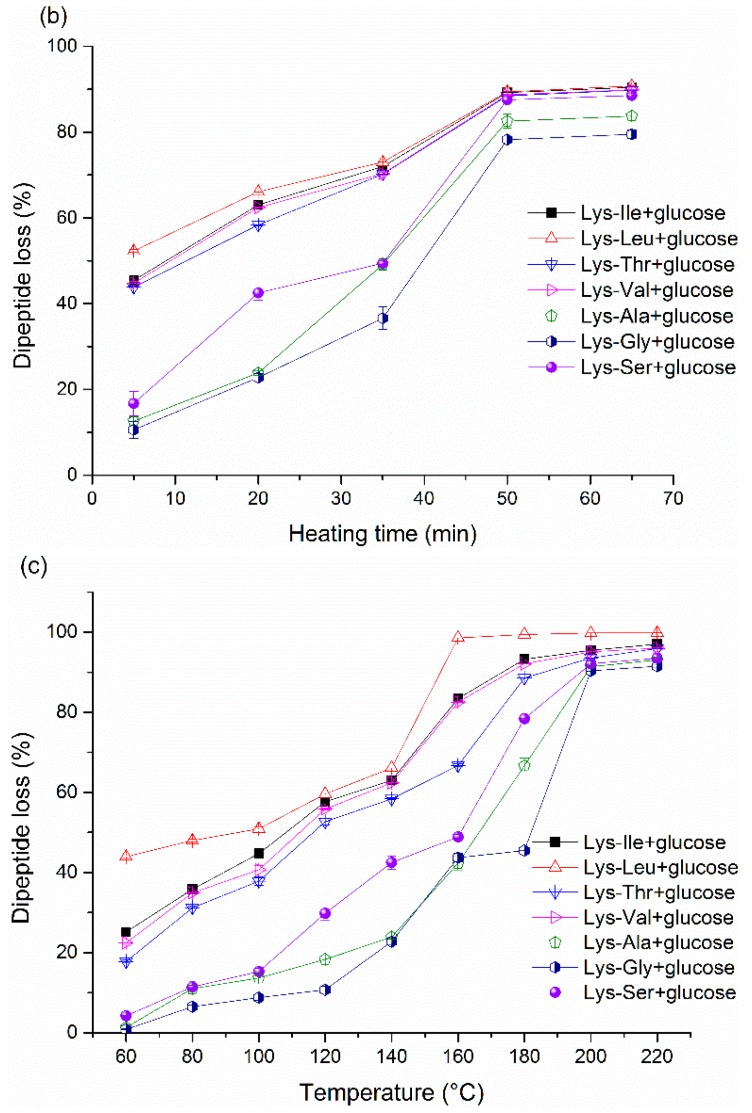
Peptide loss in the heat-treated dipeptide-glucose mixtures, (**a**) dipeptide-glucose mixtures at different initial molar ratios, time 20 min, temperature 140 °C; (**b**) dipeptide-glucose mixtures at various heat-treated times, initial molar ratio 1:1, temperature 140 °C; (**c**) dipeptide-glucose mixtures at different heating temperatures, initial molar ratio 1:1, time 20 min.

**Figure 4:**
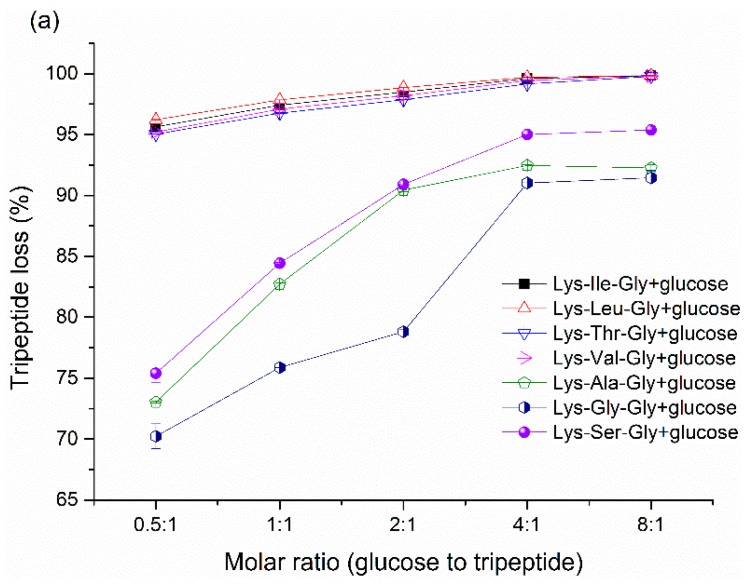

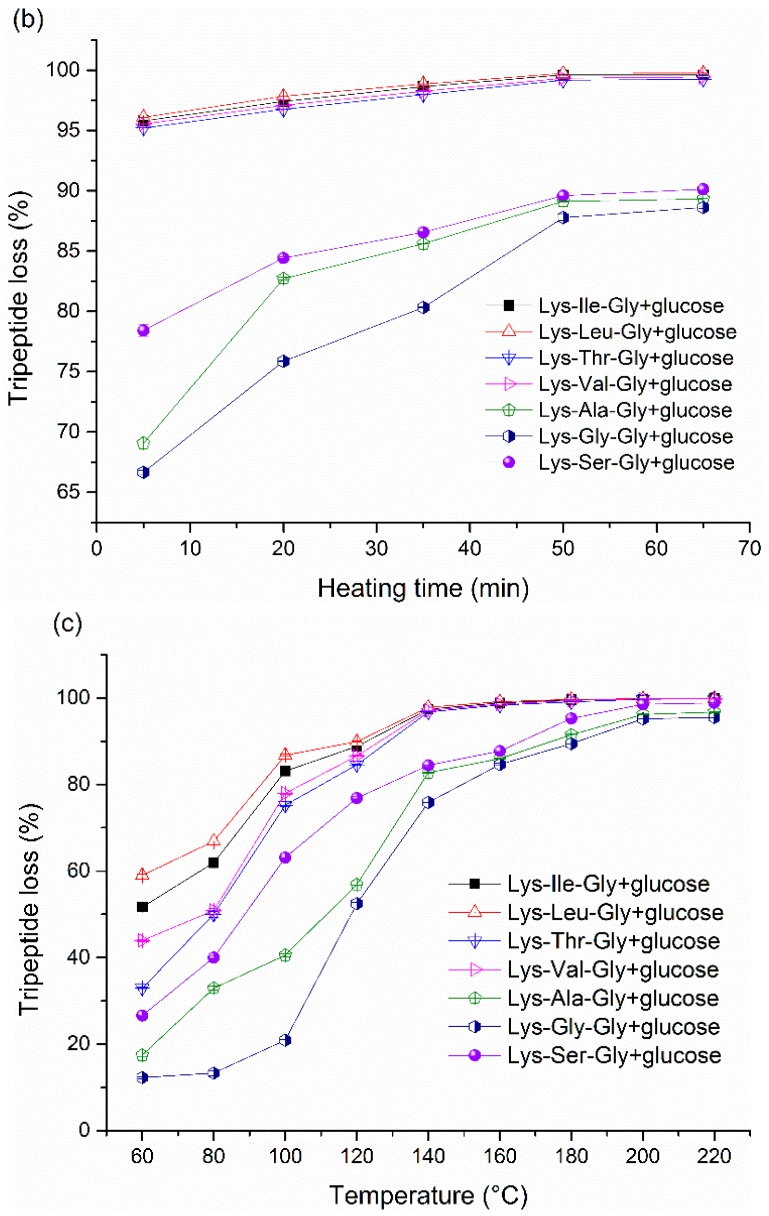
Peptide loss in the heat-treated tripeptide-glucose mixtures, (**a**) tripeptide-glucose mixtures at different initial molar ratios, time 20 min, temperature 140 °C; (**b**) tripeptide-glucose mixtures at various heat-treated times, initial molar ratio 1:1, temperature 140 °C; (**c**) tripeptide-glucose mixtures at different heating temperatures, initial molar ratio 1:1, time 20 min.

As shown in Figure 3a and Figure 4a, there was a steady increase in peptide loss with increasing molar ratios in all peptide-glucose mixtures. When the molar ratio was greater than 4:1, the peptide loss raised up to >90% in all peptide-glucose mixtures. In dipeptide-glucose mixtures, the loss of each dipeptide had the same trend with increasing molar ratios (Figure 3a). In tripeptide-glucose mixtures, tripeptide loss increased slightly in Lys-Ile-Gly/glucose, Lys-Leu-Gly/glucose, Lys-Thr-Gly/glucose and Lys-Val-Gly/glucose with increasing molar ratios, respectively (Figure 4a). However, there was no significant difference (*p* > 0.05) in tripeptide loss among these four tripeptide-glucose mixtures at the same initial molar ratio. In addition, Lys-Ala-Gly/glucose, Lys-Gly-Gly/glucose and Lys-Ser-Gly/glucose showed a substantial increase in tripeptide loss with increasing molar ratios until 4:1, respectively. The tripeptide loss in these three tripeptide-glucose mixtures decreased in the following order at the same initial molar ratio: Lys-Ser-Gly/glucose > Lys-Ala-Gly/ glucose > Lys-Gly-Gly/glucose.

The dipeptide loss *vs.* time and tripeptide loss *vs.* time are shown in the Figure 3b and Figure 4b, respectively. The trend in dipeptide loss *vs.* time in Figure 3b was similar to the trend in dipeptide loss *vs.* molar ratio in Figure 3a. Similarly, the trend in tripeptide loss *vs.* time in Figure 4b was very similar to the trend in tripeptide loss *vs.* molar in Figure 4a. Generally, the peptide loss increased steadily with increasing heating time until 50 min, both dipeptide loss and tripeptide loss were beyond 75% after 50 min.

Figure 3c and Figure 4c show the dipeptide loss *vs.* temperature and tripeptide loss *vs.* temperature respectively. Generally, both dipeptide loss and tripeptide loss increased steadily with increasing heating temperatures until 200 °C. At 200 and 220 °C, peptide loss could reach above 90%, it indicates that the vast majority of peptides are involved in the intricate reactions.

In addition, in terms of the slope of peptide loss curve (the rate of peptide loss), it was lower in dipeptide-glucose mixtures than that in tripeptide-glucose with increasing temperatures from 60 °C to 140 °C, while a reverse result was observed when temperature exceeding 140 °C.

### 2.3. Peptide Bound Pyrraline in the Mixtures

**Figure 5:**
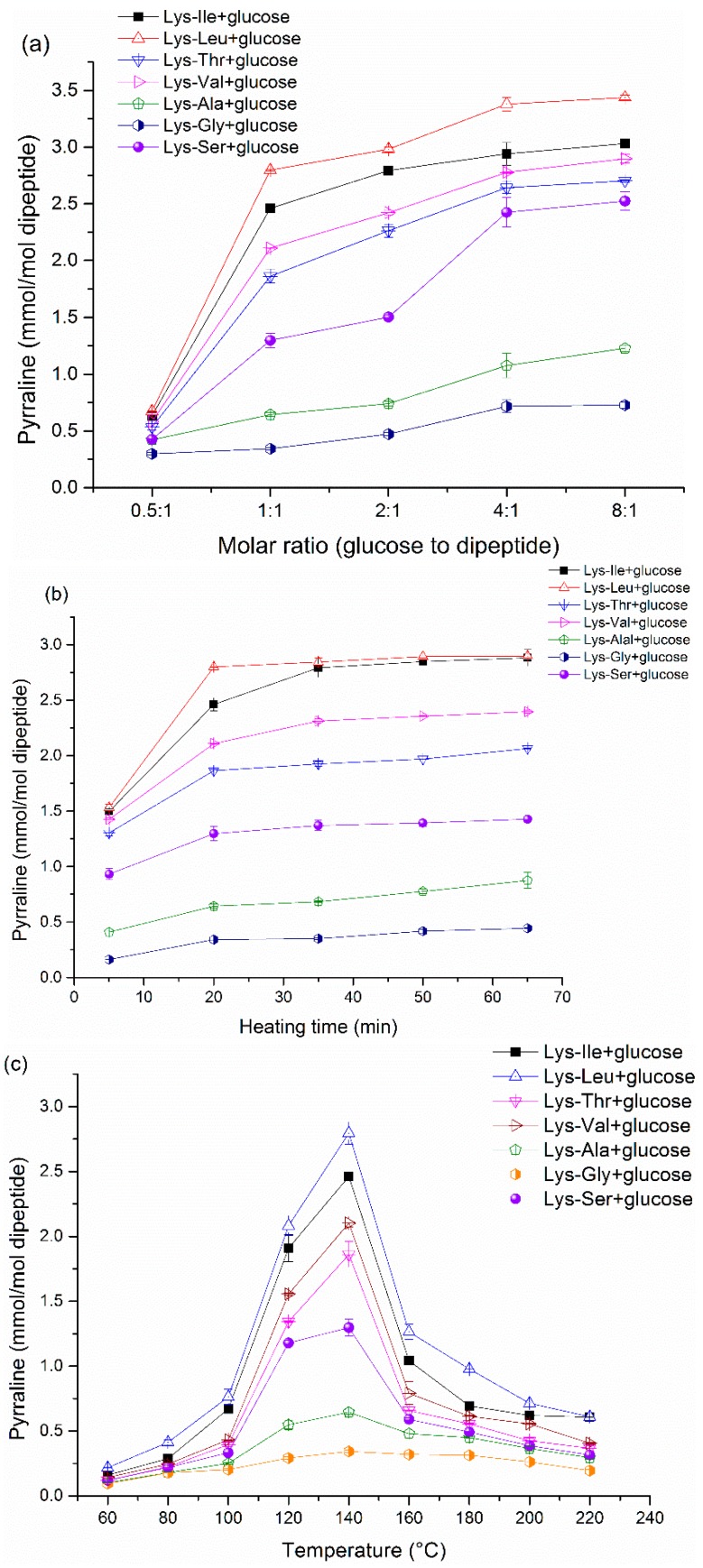
Peptide-bound pyrraline concentration in the heat-treated dipeptide-glucose mixtures, (**a**) dipeptide-glucose mixtures at different initial molar ratios, time 20 min, temperature 140 °C; (**b**) dipeptide-glucose mixtures at various heat-treated times, initial molar ratio 1:1, temperature 140 °C; (**c**) dipeptide-glucose mixtures at different heating temperatures, initial molar ratio 1:1, time 20 min.

**Figure 6:**
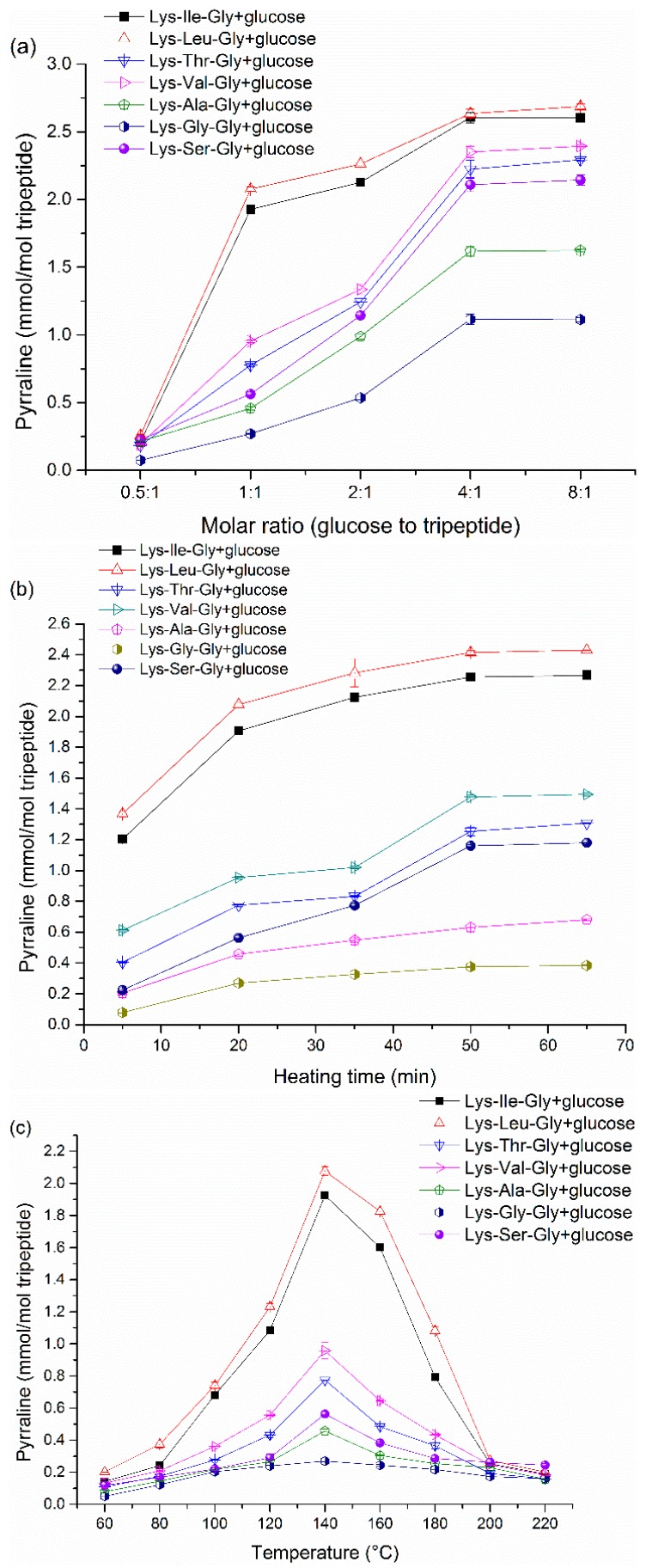
Peptide-bound pyrraline concentration in the heat-treated tripeptide-glucose mixtures, (**a**) tripeptide-glucose mixtures at different initial molar ratios, time 20 min, temperature 140 °C; (**b**) tripeptide-glucose mixtures at various heat-treated times, initial molar ratio 1:1, temperature 140 °C; (**c**) tripeptide-glucose mixtures at different heating temperatures, initial molar ratio 1:1, time 20 min.

Effect of the reactant molar ratios on the peptide bound pyrraline formation are shown in Figure 5a and Figure 6a. In dipeptide-glucose mixtures, peptide bound pyrraline content increased with increasing molar ratios in all dipeptide-glucose model systems (Figure 5a). Different concentrations of peptide-bound pyrraline were detected in various dipeptide-glucose model systems at the same molar ratio. In addition, in dipeptide-glucose model systems, dipeptide-bound pyrraline concentration (significant difference, *p* < 0.05) decreased in the following order at the same molar ratio: Pyr-Leu > Pyr-Ile > Pyr-Val > Pyr-Thr > Pyr-Ser > Pyr-Ala > Pyr-Gly. In Figure 6a, a similar result of tripeptide-bound pyrraline (Pyr-Leu-Gly > Pyr -Ile-Gly > Pyr-Val-Gly > Pyr-Thr-Gly > Pyr-Ser-Gly > Pyr-Ala-Gly > Pyr-Gly-Gly) was observed in tripeptide-glucose model systems as compared to that in the dipeptide-glucose model systems. In addition, comparing each dipeptide-bound pyrraline in Figure 5a with the corresponding tripeptide-bound pyrraline in Figure 6a at the same molar ratio, a conclusion can be drawn that the peptide-bound pyrraline is formed easier in dipeptide-glucose mixtures than that in the corresponding tripeptide-glucose mixture.

In order to study the effect of heating time on the peptide-glucose reaction, the peptide-bound pyrraline content was monitored over the time intervals (Figure 5b and Figure 6b). In Figure 5b, the dipeptide-bound pyrraline content increased slightly with increasing heating times, however, when heating time was greater than 50 min, the dipeptide-bound pyrraline content reached a plateau at 2.85 ± 0.01, 2.89 ± 0.07, 1.97 ± 0.02, 2.36 ± 0.01, 0.78 ± 0.02, 0.42 ± 0.02 and 1.39 ± 0.03 mmol/mol peptide (significant difference, *p* < 0.05) in Lys-Ile/glucose, Lys-Leu/glucose, Lys-Thr/glucose, Lys-Val/glucose, Lys-Ala/glucose, Lys-Gly/glucose and Lys-Ser/glucose model systems, respectively. In addition, comparing the peptide-bound pyrraline concentration among these seven model systems at the same heating time, the dipeptide-bound pyrraline concentration decreased in the following order: Pyr-Leu > Pyr-Ile > Pyr-Val > Pyr-Thr > Pyr-Ser > Pyr-Ala > Pyr-Gly. Likewise, there was a similar result of tripeptide-bound pyrraline in tripeptide-glucose mixtures (Figure 6b). Moreover, the results in Figure 5b and Figure 6b indicate that the concentration of each kind of peptide-bound pyrraline in dipeptide-glucose mixtures is higher than that in the corresponding tripeptide-glucose mixture.

The Maillard reaction depends greatly on temperature with respect to which reaction route prevails and what pattern of intermediates and end products are formed. The concentrations of various dipeptide-bound pyrralines and tripeptide-bound pyrralines at different temperatures are shown in Figure 5c and Figure 6c. In dipeptide-glucose mixtures, the content of peptide-bound pyrraline increased steadily with increasing temperatures until 100 °C, then ascended rapidly from 100 °C to 120 °C, and after that, increased again steadily until 140 °C (Figure 5c). At 140 °C, the maximum concentrations of each peptide-bound pyrraline were detected at 2.46 ± 0.01, 2.80 ± 0.08, 1.86 ± 0.10, 2.10 ± 0.03, 0.64 ± 0.02, 0.34 ± 0.01 and 1.30 ± 0.06 mmol/mol peptide in Lys-Ile/glucose, Lys-Leu/glucose, Lys-Thr/glucose, Lys-Val/glucose, Lys-Ala/glucose, Lys-Gly/glucose and Lys-Ser/glucose model systems, respectively. Then, there was a significant decrease in each peptide-bound pyrraline content from 140 °C to 160 °C. After 160 °C, the pyrraline content dropped steadily until 220 °C. Among these dipeptide-glucose model systems, the peptide-bound pyrraline concentration decreased in the following order at the same temperature from 100 °C to 180 °C: Pyr-Leu > Pyr-Ile > Pyr-Val > Pyr-Thr > Pyr-Ser > Pyr-Ala > Pyr-Gly. Meanwhile, an analogous result (Pyr-Leu-Gly > Pyr -Ile-Gly > Pyr-Val-Gly > Pyr-Thr-Gly > Pyr-Ser-Gly > Pyr-Ala-Gly > Pyr-Gly-Gly) was observed in tripeptide-glucose mixtures (Figure 6c). There was a steady increase in peptide-bound pyrraline content with increasing temperatures from 60 °C to 120 °C, a significant increase in peptide-bound pyrraline content was observed from 120 °C to 140 °C. The highest concentrations of peptide-bound pyrraline were detected at 140 °C (1.92 ± 0.02, 2.08 ± 0.03, 0.77 ± 0.01, 0.95 ± 0.05, 0.46 ± 0.01, 0.27 ± 0.01 and 0.56 ± 0.01 mmol/mol peptide in Lys-Ile-Gly/glucose, Lys-Leu-Gly/glucose, Lys-Thr-Gly/glucose, Lys-Val-Gly/glucose, Lys-Ala-Gly/glucose, Lys-Gly-Gly/glucose and Lys-Ser-Gly/glucose model systems, respectively). Above 140 °C, a sharp decrease was observed in all tripeptide-glucose mixtures with increasing temperatures.

### 2.4. Quantitative Structure-Reactivity Relationship (QSRR) Modelling of Peptide-Bound Pyrraline Formation

Based on the above results, the amounts of peptide bound pyrraline were different among various dipeptide-glucose mixtures or tripeptide-glucose mixtures. Therefore, a QSRR model was generated to explore the essence of this observation. According to the AAindex database [23], many researchers have reviewed the physicochemical properties of amino acids and their corresponding residues. A set of variables describing the physicochemical properties of amino acids (hydrophobicity, pK_b_, residue volume, polarizability, molecular volume and localized electrical effect) was chosen in the present study. The data of hydrophobicity and localized electrical effect were collected from literature review by Fauchere *et al.* [24]. The data of pK_b_ (α-NH_2_) were obtained from a biochemistry handbook [25]. The residue volume and molecular volume data were individually extracted from the reviews of Goldsack *et al.* [26] and Grantham [27]. The difference among either seven dipeptides or seven tripeptides is only the amino acid adjacent to lysine, therefore, it is meaningful to reveal the relationship between neighbouring side chains and the peptide reactivity in the Maillard reaction.

**Figure 7:**
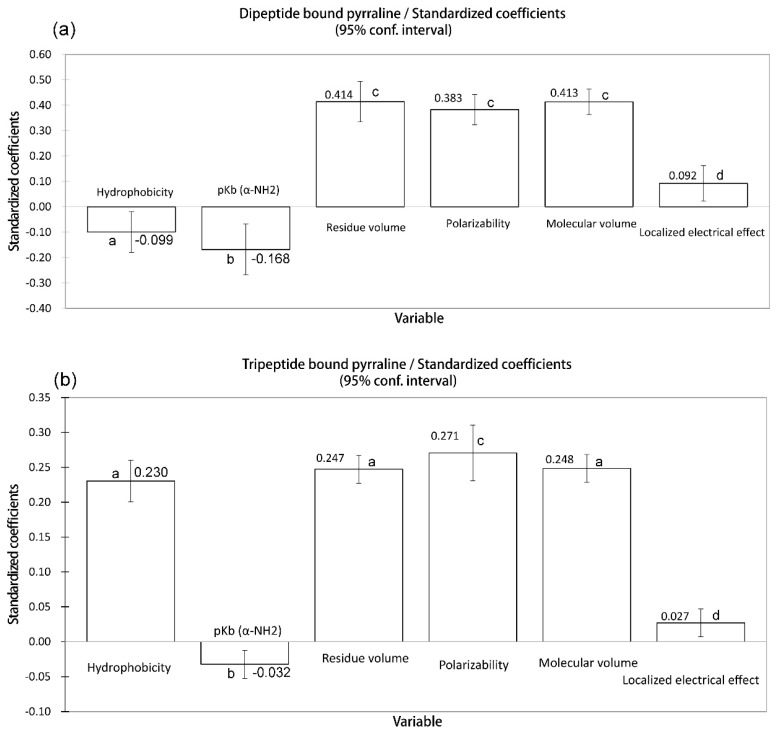
PLS regression standardized coefficients (**a**) for dipeptides and (**b**) for tripeptides. The importance of a given X-variable is proportional to its distance (coefficient value) from the origin (zero). The bars indicate 95% confidence intervals based on jack-knifing, bars with different letters have mean values that are significantly different (*p* < 0.05).

The values of physicochemical descriptors and the amounts of peptide-bound pyrraline are shown in Table 1. QSRR models of peptide bound pyrraline formation were calculated by using partial least squares regression (PLSR). The importance of a given X-variable for Y is proportional to its distance from the origin in the loading space and corresponds to the PLSR coefficients [28]. The expected descriptors of neighbouring side chain are evaluated based on their importance to the Y-variable. For dipeptides (Figure 7a), residue volume, polarizability, molecular volume and localized electrical effect were positively related to the yield of dipeptide-bound pyrraline, while hydrophobicity and pK_b_ were negatively related to the yield of dipeptide-bound pyrraline. In terms of standardized coefficients values, it was evident that residue volume, molecular volume and polarizability contributed equal and most significant impact on the yield of peptide-bound pyrraline, followed by pK_b_, then hydrophobicity and localized electrical effect.

For tripeptides (Figure 7b), hydrophobicity, residue volume, polarizability, molecular volume and localized electrical effect were positively related to yield of tripeptide-bound pyrraline, only pK_b_ was negatively related to the yield of tripeptide-bound pyrraline. In respect of standardized coefficients values, the importance of amino acid residue in yielding peptide-bound pyrraline was mainly decided by polarizability, followed by molecular volume, residue volume and hydrophobicity, then pK_b_ and localized electrical effect.

## 3. Discussion

3-DG, a dicarbonyl compound, is proved to be the main intermediate for pyrraline and browning formation [29]. Figure 1a and Figure 2a show the effect of initial molar ratio on 3-DG formation in dipeptide-glucose mixtures and tripeptide-glucose mixtures, respectively. The 3-DG concentration was very low at 0.5:1 molar ratio due to the low absolute initial content of glucose, since the mmol/mol glucose was employed as the unit of 3-DG content. Although it stands to reason that the absolute content of 3-DG increases with the increasing content of glucose, the 3-DG concentration (measured as mmol/mol glucose) decreases gradually with increasing molar ratios after above 1:1 because of the Maillard reaction product formation. It is obvious that more 3-DG is produced in the Maillard reaction with increasing glucose content. However, in terms of heating time, decreasing trends in 3-DG with increasing heating times were observed in Figure 1b and Figure 2b. During continuous heating, the high content of 3-DG at the initial stage indicates that 3-DG can be formed within short heating time, however, the 3-DG content drops gradually to form other compounds with prolonged heating conditions. In the Maillard reaction, there are two pathways to form 3-DG (Scheme 1). Firstly, 3-DG can be formed by glucose degradation [18]. Besides, 3-DG can be produced after the Amadori rearrangement via a 1,2-enolisation pathway [30]. In fact, during the prolonged heating, the amine group can essentially react with 3-DG to produce pyrraline, melanoidins *etc*. However, there was a very complicated trend in 3-DG content (Figure 1c and Figure 2c) with increasing temperatures. The 3-DG content was very low when the temperature was below 80 °C; this result may be attributed to low activation energy. Beyond 80 °C, the activation barrier can be surpassed easily. The 3-DG concentration reached a maximum at 100 °C. Simultaneously, the formation rate of peptide-bound pyrraline began to rise up when the temperature exceeded 100 °C (Figure 5c and Figure 6c). This suggests that the rapid increase in peptide-bound pyrraline concentration is mainly because of the sufficient amounts of 3-DG derived from glucose when the temperature exceeds 100 °C. However, because 3-DG can react with amine groups during the final stage of the Maillard reaction, a decrease in 3-DG content was displayed inevitably when the temperature exceeded 100 °C. Unexpectedly, 3-DG content dropped sharply at 160 °C in this declining trend, a very high formation rate of 3-DG should be determined even though there was a low content of 3-DG. Systematically considering the Maillard reaction, there are two possible reasons to account for this exception. One reason is that different kinds of nitrogen compounds can be formed by reacting dicarbonyl compounds with amine groups [31,32]. Pyrraline is just one of these heterocyclic nitrogen compounds. Some other nitrogen compounds derived from 3-DG may be produced easier than pyrraline at 160 °C, thus causing more 3-DG consumption. Another reason is that the temperature may influence the amounts and kinds of dicarbonyl compounds. Other uncertain compounds excluding 3-DG may be the predominant dicarbonyl compound at 160 °C [31]. However, further research is needed to gain deeper insight into the sharp decrease in 3-DG content at 160 °C. Generally, the 3-DG content decreased in the following order (only referring to the conditions with a significant difference, *p* < 0.05): Lys-Leu/glucose > Lys-Ile/glucose > Lys-Val/glucose > Lys-Thr/glucose > Lys-Ser/glucose > Lys-Ala/glucose > Lys-Gly/ glucose; Lys-Leu-Gly/glucose > Lys-Ile-Gly/glucose > Lys-Val-Gly/glucose > Lys-Thr-Gly/glucose > Lys-Ser-Gly/glucose > Lys-Ala-Gly/glucose > Lys-Gly-Gly/glucose. Some exceptions with no significant difference (*p* > 0.05) may be due to the very low 3-DG content and the detection limit. Besides, 3-DG can be formed easier in dipeptide-glucose mixtures than that in the corresponding tripeptide-glucose mixtures. These results suggest that the 3-DG formation from Schiff base is influenced by the amino acid adjacent to Lys and the length of peptides in the Maillard reaction. A catalytic conformation of the dipeptide/glucose adduct has been advocated by De Kok [18]. Based on De Kok’s hypothesis, by substituting the Lys-X with Lys-X-Gly (X = Ala, Gly, Ser, Ile, Leu, Thr, Val), the increase in chain length can introduce a great amount of new degrees of freedom (*i.e.*, a large number of possible effective conformations). From a statistical point of view, the possibility of the appearance of direct interaction of the COOH group with the amino terminus decreases with further increasing chain length [18].

Peptide loss is an important indicator to monitor the Maillard reaction. The peptide loss *vs.* initial molar ratio are shown in Figure 3a and Figure 4a. Peptide loss can reach above 90% with the excess of glucose, it indicates that the vast majority of peptide can be involved in the Maillard reaction with sufficient glucose. However, correlating peptide loss and peptide-bound pyrraline formation, it indicates that higher peptide loss does not imply a higher content of pyrraline formation (Figure 3c, Figure 4c, Figure 5c and Figure 6c). Although pyrraline is derived from the reaction between amine group and glucose, large amounts of other products derived from aldehyde-amine condensation can also be produced at the final stage of the Maillard reaction. Unfortunately, it is very difficult to classify the final products with the dark-brown color formed in foods, since they tend to be very complex mixtures and are chemically relatively intractable [33].

Because the peptide-bound pyrraline is the main target in the present study, thus the formation of peptide-bound pyrraline needs to be elucidated carefully and in detail. It is easy to chemically understand that the content of peptide-bound pyrraline increases with increasing initial molar ratios. Generally, more initial amounts of reactants can lead to higher content of peptide-bound pyrraline formation. In addition, there is a similar trend in peptide-bound pyrraline concentration over heating time. The final content of peptide-bound pyrraline reaches a plateau with a continuous increase in initial molar or heating time. It can be explained that the formation of peptide-bound pyrraline is a dynamic reaction process [34], the product may reach a equilibration after prolonged heating time, as well as with an excess of one of the reactants. The maximum amounts of peptide-bound pyrraline were produced at 140 °C. There was a gradual increase in peptide-bound pyrraline content with increasing temperatures below 140 °C, but an inverse trend was observed when the temperature exceeded 140 °C. It is well known that the Maillard reaction is a systematic and complex reaction, and pyrraline formation is not the only route of the Maillard reaction. In terms of reaction equilibrium of peptide-bound pyrraline formation, the peptide-bound pyrraline formation (forward reaction) may be the predominant reaction when the temperature is below 140 °C compared with the reverse reaction. A reverse result was obtained at temperatures above 140 °C due to the formation of pyrraline elimination products (*i.e.*, a dipyrraline and a crosslinked product formed by pyrraline reacting with lysine) [34]. Briefly, these results are caused by the comprehensive effects of various factors in the Maillard reaction.

In the present study, the results of the reaction between glucose and Lys-containing dipeptides or tripeptides have shown that the reactivity of Lys residues is greatly affected by the side chain of neighboring amino acids. The dipeptide-glucose model system produces high amounts of peptide-bound pyrraline as compared to the corresponding tripeptide-glucose model system. Lancker has mentioned that the pyrazine production from tripeptides is lower than that from dipeptides [35]. Therefore, the results of peptide-bound pyrraline production in the present study are consistent with pyrazine production as described by Lancker. More importantly, peptide-bound pyrraline formation is influenced by the peptide species under the same reaction conditions. This indicates that although the pyrrole ring of pyrraline is formed from the ε-NH_2_ of Lys residues, the neighboring amino acid can influence the ε-NH_2_ reactivity of Lys residues. Therefore, a conclusion can be drawn that the reactivity of Lys-containing peptides in peptide-bound pyrraline formation is related to the physico-chemical properties of the amino acid adjacent to Lys.

The importance of hydrophobic interactions has long been recognized in various facets of science [36]. Otto and Engberts have described the influence of hydrophobic interactions on chemical reactivity. The influence of hydrophobic interaction can be harnessed to speed up organic reactions in water [37]. In Table 1, a higher content of peptide-bound pyrraline is seen in the mixtures containing Ile or Leu residues than in the mixtures containing other amino acids. Peptides containing Ile or Leu side chain exhibited high hydrophobicity as compared to peptides containing Gly or Ser (Table 1). Consequently, there is an accelerating potential of pyrraline formation with hydrophobic side chains adjacent to Lys.

In the present study, the importance of polarizability in the reactivity of peptides in the Maillard reaction was noteworthy. The peptide-bound pyrraline formation from different peptides is influenced by polarizability of the amino acid adjacent to Lys (Table 1 and Figure 7). It has been found that the polarizability is proportional to the cubic power of the softness [38,39,40,41], and polarizability is also a quantitative indicator defined in chemical reactivity to understand the propensity of a molecule to react as a nucleophile. This fact also paves the way to propose a “minimum polarizability principle” which states that the natural direction of evolution of any system is toward a state of minimum polarizability [42]. During the initial stage of the Maillard reaction, sugar-amine condensation and Amadori rearrangement, these reactions occur between the reactive carbonyl groups of sugars and the nucleophilic amine groups of amino acids. Therefore, in the peptide-bound pyrraline formation pathway, when the Schiff base precursors are located in close proximity to a group with proton abstraction capability, the polarizability of the neighboring amino acid may play a key role in determining the rate of formation of Schiff base adducts. In addition, polarizability has been recognized as an important factor in determining nucleophilicity [43,44,45]. Based on Pearson’s concept of hard and soft acids or bases, the so-called HSAB principle [46], very polarizable peptides (*i.e.*, Lys-Leu, Lys-Ile; Lys-Leu-Gly, Lys-Ile-Gly) are highly reactive toward polarizable substrates (the carbonyl group of glucose), while poorly polarizable peptides (*i.e.*, Lys-Gly; Lys-Gly-Gly) show a low reactivity (Table 1). Consequently, a preliminary conclusion can be drawn that 3-DG production derived from Schiff bases is also influenced by the difference in polarizability. Besides, peptide-bound pyrraline is formed from peptides which react with 3-DG during the last stage (Scheme 1), this stage may also be affected by the polarizabilities of various peptides, as well as sugar-amine condensation at the initial stage of the Maillard reaction. Essentially, the side chain of amino acids may affect the electron density of the main chain atoms as a function of their physicochemical properties.

The distinction among these seven dipeptides or seven tripeptides is the side chain of the amino acid adjacent to Lys, and theoretically, there is a positive and close correlation between residue volume and molecular volume. The molecular volume is an important index of steric effects. Taft has developed the “steric” index, which attempts to associate the extent to which substituents around a reaction center hinder the reactivity of attaching groups [47]. In fact, both the α-NH_2_ and ε-NH_2_ of Lys can be involved in the Maillard reaction, it can be expected that the reactivity of the α-NH_2_ is much higher than the reactivity of the ε-NH_2_, however, according to the structure of pyrraline, the peptide-bound pyrraline is mainly formed by the reaction between the ε-NH_2_ of Lys and 3-DG [12]. Therefore, the reactivity of the α-NH_2_ of Lys may be greatly hindered in the structures of Lys-Leu, Lys-Ile, Lys-Leu-Gly and Lys-Ile-Gly with large side chain volumes. The reactivity of ε-NH_2_ is less influenced by steric effects than that of α-NH_2_, because there is a long distance between the side chain of the neighbouring amino acid and the ε-NH_2_ as compared to that in α-NH_2_. Consequently, the probability of peptide-bound pyrraline formation in Leu or Ile-containing peptide-glucose mixtures is higher than that in other amino acid-containing peptide-glucose mixtures. In addition, because peptide-bound pyrraline is mainly derived from the ε-NH_2_ of Lys, side chains with large volume, such as the side chain of Leu or Ile, can be conducive to high stability of the peptide backbone chain. In fact, a side chain with large volume can reduce the probability of peptide chain degradation in the Maillard reaction with high temperature treatment [48]. Consequently, a preliminary conclusion can be drawn that the peptides with large side chain volume adjacent to Lys may have a high probability of peptide-bound pyrraline production.

Steric effects are often contrasted to and complemented by electronic effects. The electrical effect is highly related to the induction effect, conjunction effect, orbital symmetry, electrostatic interactions, and spin state. Actually, pK_b_ is mainly influenced by delocalized electrons. Consequently, a localized electrical effect is essential for pK_b_ value. In the present study, the amino acids adjacent to Lys with lower localized (field) electrical effect or higher delocalized electrical effect such as Ala, Leu, Ile (Table 1) may exhibit higher electrical property activity. Therefore, the lower value of localized (field) electrical effect is conducive to a higher peptide reactivity in peptide-bound pyrraline formation. It also leads to the result that the yield of peptide-bound pyrraline is negatively related to pK_b_, but is positively related to localized electrical effect (Figure 7a,b). However, it is important to note that these six physicochemical properties are inherently related, the effect of physicochemical properties on peptide-bound pyrraline formation is a comprehensive result.

## 4. Materials and Methods

### 4.1. Chemicals

All chemicals used were of analytical grade unless otherwise stated. Carboxypeptidase A (from bovine pancereas), Fmoc chloride (for HPLC derivatization, ≥99.0%), D-fructose (purity 99%) and D-glucose (purity 99%), were purchased from Sigma-Aldrich (Shanghai, China). Dipeptides (Lys-Ala, Lys-Gly, Lys-Ser, Lys-Ile, Lys-Leu, Lys-Thr, Lys-Val, purity > 98%), and the corresponding tripeptides (Lys-Ala-Gly, Lys-Gly-Gly, Lys-Ser-Gly, Lys-Ile-Gly, Lys-Leu-Gly, Lys-Thr-Gly, Lys-Val-Gly, purity > 98%) were supplied by Bootech BioScience &Technology Co., Ltd. (Shanghai, China). Acetonitrile and formic acid were HPLC grade from Merck (Darmstadt, Germany). A Cleanert PEP-2 (200 mg/6 mL, Bonna-Agela Technologies Inc., Tianjin, China) solid-phase extraction (SPE) cartridge was used for purification of the Maillard reaction products. 3-Deoxyglucosone (3-DG, purity > 99.99%) was obtained from Toronto Research Chemicals (Toronto, ON, Canada). Pyrraline standard sample (purity > 99.99%) was purchased from PolyPeptide Laboratories (San Diego, CA, USA).

### 4.2. Preparation of Peptide-Glucose Model Food Systems

Each peptide (Dipeptide: Lys-Ala, Lys-Gly, Lys-Ser, Lys-Ile, Lys-Leu, Lys-Thr, Lys-Val. Tripeptide: Lys-Ala-Gly, Lys-Gly-Gly, Lys-Ser-Gly, Lys-Ile-Gly, Lys-Leu-Gly, Lys-Thr-Gly, Lys-Val-Gly) and glucose were individually added to 15 mL phosphate buffer solution (PBS, 0.2 M, pH 6.8). The peptide concentration was kept at 1 mM, the concentrations of glucose were carefully designed so that initial molar ratios of glucose to peptide were 0.5:1, 1:1, 2:1, 4:1 and 8:1, respectively.

### 4.3. Thermal Treatments and the Termination of Reaction

An aliquot of peptide-glucose mixture solution (15 mL) was placed in a vessel, sealed, and heated in a microwave heating system (Ethos Series Microwave Lab Stations, Milestone Inc., Shelton, CT, USA) with different time-temperature protocols as follows: temperatures 60, 80, 100, 120, 140, 160, 180, 200 and 220 °C; times 5, 20, 35, 50 and 65 min. When the microwave heating system was working, the fluctuation ranges of temperature and pressure inside the vessel were less than ±1 °C and ±1 bar, respectively. After the designed thermal treatments were completed, *Ο*-phenylenediamine (OPD, five fold the glucose initial molar concentration) was added immediately in order to terminate the reaction by derivatization of the 1,2-dicarbonyl compounds in the model systems to azines (e.g., 3-DG quinoxaline, abbreviated as 3-DG_qx_). The mixture was kept in the dark overnight and membrane filtered (0.45 μm), then the mixture samples were stored at −18 °C before the SPE procedure and chromatographic analysis.

### 4.4. SPE and Hydrolysis of Peptide Bound Pyrraline

A SPE cartridge was used to pretreat the mixtures containing peptide-bound pyrraline. The cartridge was preconditioned with 4 mL of methanol, and equilibrated with 4 mL of water before loading the sample. One milliliter of mixture sample was then applied to the cartridge, followed by washing with 2 mL of water. Finally, the target compounds were eluted from the cartridge with 4 mL of acetonitrile, and the eluent was evaporated to dryness at 50 °C by a gas pressure blowing concentrator. The dried residue was then dissolved in 1 mL of HPLC eluent (0.1% formic acid in water containing 15% acetonitrile) and membrane filtered (0.45 μm) before qualitative detection.

In terms of peptide-bound pyrraline quantification, samples without SPE pretreatment were subjected to carboxypeptidase A hydrolysis after adjusting pH value to 7.4 by adding sodium hydroxide solution. The sample mixtures were incubated for 3 h in the presence of 4 U/mL carboxypeptidase A at 37.5 °C, thus free pyrraline was released from peptide-bound pyrraline by the hydrolysis. The hydrolysis procedure in the present study was a complete hydrolysis procedure with high hydrolysis efficiency (See Supplementary Table S1). The supernatant was collected after centrifugation (10,000 rpm, 25 min), and then subjected to the SPE procedure as described previously. After SPE, the samples was subjected to HPLC analysis. The content of free-form pyrraline was employed to quantify the content of peptide-bound pyrraline.

### 4.5. Peptide Content Analysis

Fmoc reagent was freshly prepared from 200 mg Fmoc chloride in 100 mL acetone. For the derivatization reaction 10–100 μL samples containing unreacted peptide were added to 100 mL of PBS buffer (pH 6.8) and 700–1000 μL of the Fmoc-reagent and mixed well [49]. After 45 s the derivatization reaction was completed. The mixture was extracted two times with 2 mL of pentane/ethyl acetate (80:20). The aqueous phase containing the Fmoc derivatives was analyzed by C18 analysis column interfaced with an ESI mass spectrometer. The unreacted peptides were derivatized to (N_α_,N_ε_-di-) Fmoc derivatives in order to obtain a high response in mass spectrometer.

The peptide loss in the mixtures was calculated as {[pep]-[unreacted pep]}/[pep], [unreacted pep] was the concentration of unreacted peptide, [pep] was the initial concentration of peptide.

### 4.6. Chromatographic Procedure and Instruments

Qualitative detection of pyrraline and 3-DG in the mixture samples were assayed by an UPLC-Q-TOF system. The system was an Agilent 1290 system (Agilent Technologies, Inc., Palo Alto, CA, USA) coupled to Bruker microTOF-q II mass spectrometer (Bruker Daltonics GmbH, Bremen, Germany). The chromatographic column was an Agilent ZORBAX SB-C18 column (150 mm × 2.1 mm, 5 μm), and the temperature was set to 30 °C. The injection volume was 5 μL, mobile phase solvents consisted of 0.1% formic acid in water (A) and 0.1% formic acid in acetonitrile (B). The gradient conditions were 5% B (0 min), 5% B (0.8 min), 40% B (8 min), 5% B (10 min), 5% B (12 min). The flow rate was 0.2 mL/min.

The ESI source conditions were as follows: endplate off, −500 V; capillary voltage, 4.5 kV; nebulizer pressure, 0.3 bar; dry gas flow, 4.0 L/min; and dry temperature, 180 °C. Mass scan range was 50 to 1000 *m*/*z* in positive mode. Sum formula generation was processed by SmartFormula3D and the FragmentExplorer (Bruker Daltonics GmbH), and fragment structures were assigned by Data Analysis 4.1 (Bruker Daltonics GmbH). Profile Analysis 2.1 (Bruker Daltonics GmbH) was used for statistical data evaluation. Free-form pyrraline released from peptide bound pyrraline was quantified by its parent ion peak (*m*/*z* = 255.1300), 3-DG was determined by the parent ion peak of 3-DG_qx_ (*m*/*z* = 235.1040). External calibration was performed with standards.

### 4.7. Polarizabilities of Amino Acid Residues Calculation

All polarizability parameters calculations were performed by Gaussian 09 program [50]. Molecular geometries were previously optimized at the DFT-B3LYP level with the 6-31G∗ basis set [51]. Polarizabilities were then calculated at the same level of theory using the standard Gaussian 09 keyword “Polar”. It represented that the values of polarizability were obtained analytically rather than by numerical differentiation.

### 4.8. Partial Least Squares Regression (PLSR)

PLSR analysis and statistical test of regression coefficient were carried out using XLSTAT-PLS version 2015 software (Addinsoft SARL, Belmont, MA, USA). All variables were centered and scaled to unit variance prior to the analyses except with specification, and thereby, all variables had an equal participation in the model.

### 4.9. Statistical Analysis

All experimental data were analyzed by of variance (ANOVA) and significant differences among means from triplicate analyses (*p* < 0.05) were determined by Duncan’s multiple range tests using the statistical analysis system (SPSS 12.0 for windows, SPSS Inc., Chicago, IL, USA).

## 5. Conclusions

3-DG content in dipeptide-glucose model system was higher than that in the corresponding tripeptide-glucose model system. Dipeptides produced higher amounts of peptide-bound pyrraline than the corresponding tripeptides. The peptide-bound pyrraline and 3-DG production in peptide-glucose model systems were influenced by the side chains of amino acids adjacent to Lys in the following order: Lys-Leu/glucose > Lys-Ile/glucose > Lys-Val/glucose > Lys-Thr/glucose > Lys-Ser/glucose > Lys-Ala/glucose > Lys-Gly/glucose; Lys-Leu-Gly/glucose > Lys-Ile-Gly/glucose > Lys-Val-Gly/glucose > Lys-Thr-Gly/glucose > Lys-Ser-Gly/glucose > Lys-Ala-Gly/glucose > Lys-Gly-Gly/ glucose. This fact was caused by the physicochemical properties of the side chains of the amino acids adjacent to Lys. For side chains of amino acids adjacent to Lys in dipeptides, residue volume, polarizability, molecular volume and localized electrical effect were positively related to the yield of peptide-bound pyrraline, while hydrophobicity and pK_b_ were negatively related to the yield of peptide-bound pyrraline. In terms of the side chains of amino acids adjacent to Lys in tripeptides, the result was similar, except hydrophobicity was positively related to the yield of peptide-bound pyrraline.

## Figures and Tables

**Table 1:** Side chain physicochemical parameters (6-parameters set) for the amino acids adjacent to lysine and the yield of peptide bound pyrraline.

Adjacent Amino Acid of Lysine	Side Chain R	Hydrophobicity	Polarizability	Residue Volume	pK_b_^α^	Localized Electrical Effect	Molecular Volume	Dipeptide Bound Pyrraline ^β^	Tripeptide Bound Pyrraline ^γ^
Ala	CH_3_	0.31	50.16	88.3	9.71	−0.01	31	0.64	0.46
Gly	H	0.00	36.66	60.0	9.58	0.00	3	0.34	0.27
Ser	CH_2_OH	−0.04	53.82	88.7	9.05	0.11	32	1.30	0.56
Ile	CH(CH_3_)CH_2_CH_3_	1.80	91.21	168.5	9.60	−0.01	111	2.46	1.92
Leu	CH_2_CH(CH_3_)_2_	1.70	91.60	168.5	9.58	−0.01	111	2.80	2.08
Thr	CH(OH)CH_3_	0.26	66.46	118.2	8.96	0.04	61	1.86	0.77
Val	CH(CH_3_)_2_	1.22	76.09	141.4	9.52	0.01	84	2.10	0.96

^α^ pK_b_ (α-NH_2_); ^β^ Content of dipeptide-bound pyrraline (mmol/mol dipeptide), each dipeptide (1 mM) mixed with glucose (1 mM) in the PBS (0.2 M, pH 6.8) heated for 20 min at 140 °C; ^γ^ Content of tripeptide-bound pyrraline (mmol/mol tripeptide), each tripeptide (1 mM) mixed with glucose (1 mM) in the PBS (0.2 M, pH 6.8) heated for 20 min at 140 °C.

## Supplementary Materials

Supplementary materials can be accessed at: http://www.mdpi.com/1420-3049/21/4/463/s1.
