# Supplementary material for: Formation of Peptide Bound Pyrraline in the Maillard Model Systems with Different Lys-Containing Dipeptides and Tripeptides
Source: Molecules. 2016 Apr 7;21(4):463. doi: 10.3390/molecules21040463 (PMC6274133; doi:10.3390/molecules21040463)
Supplement: Supplementary file 1 [file molecules-21-00463-s001.pdf]

Supplementary Materials: Formation of Peptide Bound Pyrraline in the Maillard Model Systems with Different Lys-Containing Dipeptides and Tripeptides

Zhili Liang, Lin Li, Haiping Qi, Liting Wan, Panfu Cai, Zhenbo Xu and Bing Li

Table S1. The hydrolysis efficiency $^{\alpha}$ of carboxypeptidase A in different peptide-glucose model systems.

**Model Systems** $^{\beta}$	**Hydrolysis Efficiency** $^{\alpha}$ **(%)**	**Model Systems** $^{\beta}$	**Hydrolysis Efficiency** $^{\alpha}$ **(%)**
Lys-Ala + glucose	98.3 ± 0.3	Lys-Ala-Gly + glucose	94.6 ± 1.6
Lys-Gly + glucose	94.7 ± 1.2	Lys-Gly-Gly + glucose	96.8 ± 1.5
Lys-Ser + glucose	97.3 ± 0.6	Lys-Ser-Gly + glucose	95.3 ± 0.6
Lys-Ile + glucose	96.8 ± 0.9	Lys-Ile-Gly + glucose	96.7 ± 0.9
Lys-Leu + glucose	95.9 ± 1.1	Lys-Leu-Gly + glucose	94.2 ± 1.3
Lys-Thr + glucose	96.3 ± 1.4	Lys-Thr-Gly + glucose	93.5 ± 1.8
Lys-Val + glucose	97.2 ± 1.0	Lys-Val-Gly + glucose	94.2 ± 2.1

$^{\alpha}$ Hydrolysis efficiency $= \frac{Concentration\ of\ free\ Lys\ after\ incubation}{Initial\ concentration\ of\ peptide} \times 100\%$; $^{\beta}$ Both peptides and glucose concentrations were 1 mM in PBS buffer (pH 7.4) without thermal treatment, incubated in the presence of 4 U/mL carboxypeptidase A at 37.5 °C for 3 h.
